# Roussouly type 2 could evolve into type 1 shape as sagittal spinal alignment deterioration progresses with age

**DOI:** 10.3389/fsurg.2022.1049020

**Published:** 2022-11-09

**Authors:** Wenzhi Sun, Yongjin Li, Xiaolong Chen, Baobao Wang, Chao Kong, Peng Wang, Shibao Lu

**Affiliations:** Department of Orthopaedics, Capital Medical University XuanWu Hospital, Beijing, China

**Keywords:** cross-sectional study roussouly classification, sagittal alignment, elderly, degenerative, scoliosis

## Abstract

**Study design:**

Cross-sectional study.

**Objective:**

To identify whether Roussouly type 2 could evolve into type 1 as the deterioration progresses.

**Methods:**

The study group comprised subjects with a low pelvic incidence (PI). All subjects underwent a standing whole spinal radiograph and sagittal parameters were measured: T1 pelvic angle (TPA), lumbar lordosis (LL), PI, pelvic tilt (PT), L4–S1 angle, thoracolumbar kyphosis (TLK), thoracic kyphosis (TK), lumbar sagittal apex (LSA), lordosis distribution index (LDI) and number of vertebrae included in the lordosis (NVL). All subjects were distributed into two groups; with primary (*de novo*) degenerative scoliosis (PDS) and without PDS. Subjects without PDS were divided into young adult, adult, middle-aged and elderly groups. The differences in sagittal parameters of each subgroup were compared.

**Results:**

In total, 270 subjects were included with a mean age of 58.6 years (range 20–87 years). There was a stepwise increase in the proportion of type 1 with age, whereas type 2 decreased. The TPA, PT, PI-LL, TK, TLK and LDI increased with age in subjects without PDS. The TPA, LDI, TLK and TK increased with age in subjects who displayed type 1, whereas the PT, LL, L4–S1 and PI-LL were unchanged. The TPA, PT, PI-LL and TLK increased with age in subjects who displayed type 2, whereas LL and L4-S1 were decreased, while the LDI and TK remained unchanged. The LSA of subjects without PDS became lower and the NVL decreased with age, with similar phenomena found in the subjects with type 2. There was no statistical difference among the groups for the LSA or NVL distribution of subjects with type 1. The TPA, PT and PI-LL of subjects with PDS were greater than those in Group IV, while the SS, LL and TK were less. The Roussouly-type, NVL and LSA distribution were identical between these two groups.

**Conclusion:**

Roussouly type 1 shape may not be an actual individual specific spine type. Rather, type 2 could evolve into the “type 1” shape as deterioration of the sagittal spinal alignment progresses with age. Primary (*de novo*) degenerative scoliosis had little effect on whether type 2 became type 1. This should be taken into consideration during the assessment and restoration of sagittal balance.

## Introduction

Sagittal balance of the spine is a recent and ever more common viewpoint for understanding and treating spinal pathologies (1). In 2005, Roussouly et al. presented a classification based on the spinal shapes in the normal population (2). In the Roussouly classification, four classical types of spinal alignments were described depending on the sacral slope (SS) and the shape of lumbar lordosis (LL). However, degenerative spinal disease that affects the lumbar spine decreases LL (3), and this change modifies the SS due to the need for pelvic compensation to maintain the sagittal balance (4). Therefore, the basic criterion used to classify the sagittal profile of these patients (the SS) has been substituted by the pelvic incidence (PI), which is considered to be a constant parameter through adulthood independently of pelvic compensation (5–7).

Subjects with a low PI can present with either type 1 or 2 (2, 6, 8). Type 1 appears as a long thoracolumbar kyphosis (TLK) and a short lumbar lordotic curve; the lumbar spine of type 2 has a flat back appearance (2). There are also some factors that aid in determining the sagittal shape, including the lumbar sagittal apex (LSA), the number of vertebrae included in the lordosis (NVL) and the level of the inflexion point (IP) that dictates the transition between thoracic kyphosis (TK) and LL (1, 2, 4).

**Figure 1:**
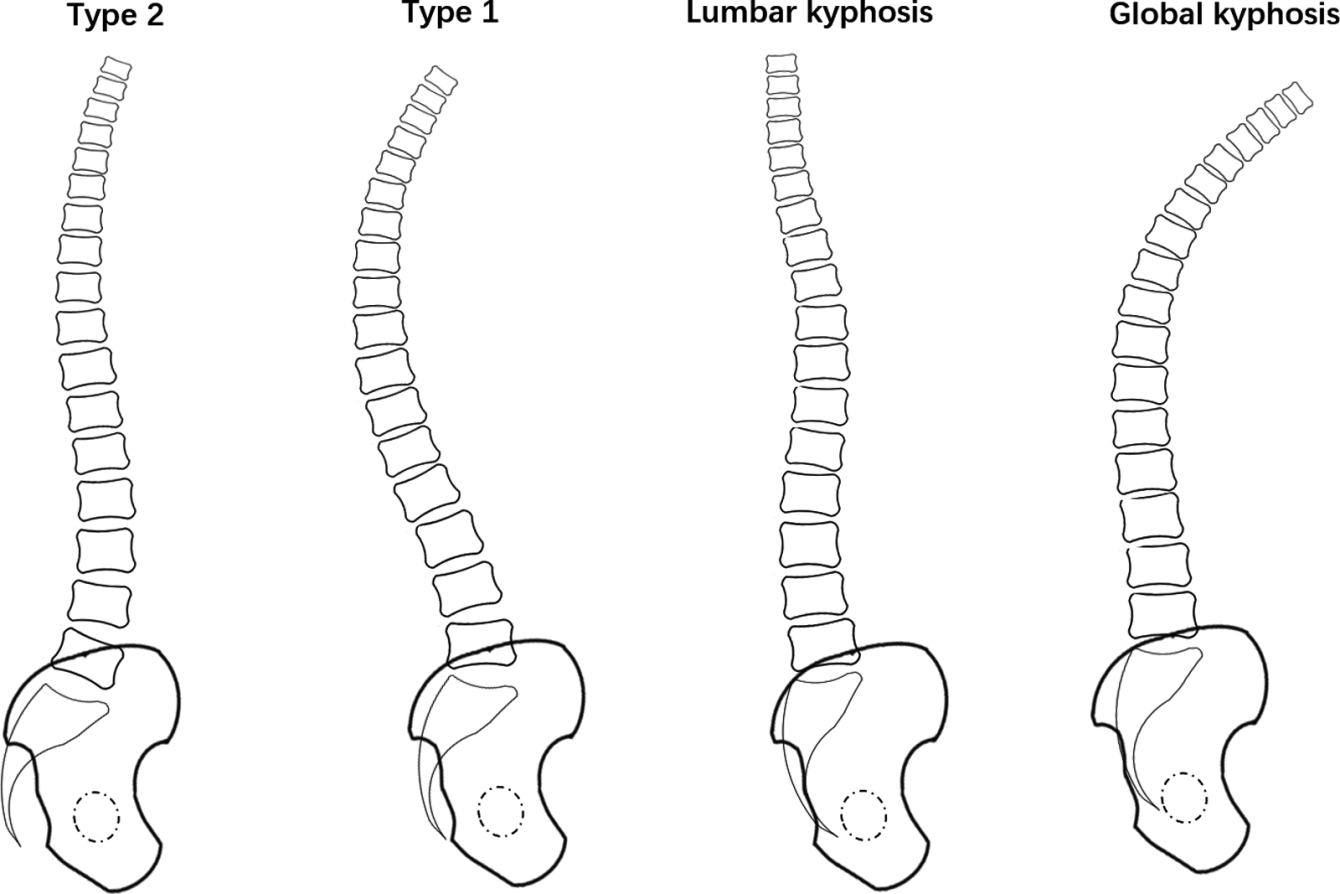
Drawing showing the possible evolution of type 2 shape. When the kyphosing event affects the thoracolumbar area or the lumbar area, either LL increases on a small arch, generating a type 1 spine, or lumbar spine lordosis resolves, generating the “lumbar kyphosis” type (if the thoracic spine could compensate with a hypokyphosis) or “global kyphosis type”.

Life is a kyphosing event. Compensation potential depends greatly on the PI; low PI types have little compensation potential, whereas high PI types have greater potential, with type 4 having the greatest potential for compensation (8). When the kyphosing event affects the thoracolumbar area or the lumbar area, either LL increases on a small arch, generating a type 1 spine, or lumbar spine lordosis resolves, generating the “lumbar kyphosis” type (if the thoracic spine could compensate with a hypokyphosis) or “global kyphosis type” (8) (Figure 1). Roussouly et al. (8) hypothesized that type 1 could be a degenerative evolution of type 2. Therefore, the type 1 shape may not be an actual individual specific spine type. Nevertheless, this theory has to date not been supported by any radiological measurement study. In the current study, we aimed to examine the radiological characteristics of the Roussouly types with a PI ≤ 50° to identify whether type 2 could evolve into type 1 with the progress of deterioration.

## Materials and methods

### Study design

This cross-sectional study was approved by the relevant institutional Ethics Committee. We informed all the subjects about the purposes, methods and risks of the study, and subsequently they provided written informed consent before their enrollment.

### Subject recruitment

On the basis of the following inclusion and exclusion criteria, 270 subjects were recruited in the present study: Inclusion criteria included: (1) age ≥20 years, (2) PI ≤ 50°. Exclusion criteria were: (1) subjects who had already undergone spinal surgery; (2) history of trauma, tumor or infection of the spine; (3) lumbosacral transitional vertebrae; (4) neuromuscular disease; (5) acute pain or any other condition that may affect the accurate measurement of radiological parameters; (6) subjects with scoliosis except primary (*de novo*) degenerative scoliosis (PDS); (7) subjects with lumbar kyphosis or global spine kyphosis.

### Radiographic measurements

All the subjects underwent full-length lateral and antero-posterior x-rays, including of their hip joints. All radiographs were analyzed using validated software (Surgimap, Nemaris Inc., New York, NY).

The following spinal and pelvic radiographic parameters were measured: Pelvic parameters consisted of the PI, pelvic tilt (PT) and SS. Spinal parameters included LL (Cobb angle between the superior endplate of L1 and S1), L4–S1 angle Cobb angle between the upper endplate of L4 and the sacral endplate), TLK (Cobb angle between the superior endplate of T10 and the inferior endplate of L2), TK (Cobb angle between the superior endplate of T5 and the inferior endplate of T12), NVL, LSA and IP. With LL and the L4–S1 angle, the percentage L4–S1 contribution to the total lordosis was calculated and termed the lordosis distribution index (LDI) (9). Lumbar mismatch was calculated as the PI-LL. Global sagittal balance was evaluated using the T1 pelvic angle (TPA, the angle formed by the line from the center of T1 to the femoral head axis and the line from the center of the sacral endplate to the femoral head axis (10)).

Coronal parameters were also assessed: thoracolumbar coronal (TLC) Cobb angle, lumbar-sacrum coronal (LSC) Cobb angle and the apical vertebra rotation (AVR) of the thoracolumbar curve. The Nash–Moe classification (Grades 0–IV; the higher the grade, the more severe the vertebral rotation degree) was determined, which reflected the degree of vertebral rotation (11).

The classical type 1 was defined by a long TLK, a short lumbar lordotic curve and a PI ≤ 50°; classical type 2 was defined by a long and flat lordosis and a PI ≤ 50° (2, 12). Two independent examiners (B.B.W and Y.J.L) determined the classification twice, with an interval of 1 week. Disagreements were resolved through discussion until a consensus opinion was reached. The ideal LSA of type 2 was L4/5 and the ideal LSA of type 1 was L5 (12). For statistical weight, the LSA were defined: 1 for “LSA above L4/5″, 2 for “LSA located at L4/5″ and 3 for “LSA below L4/5″.

### Statistical analysis

All the data were collected in Microsoft Excel 2019, and statistical analysis was performed using the SPSS 21.0 software (SPSS Inc., Chicago, IL, United States). The variables were described as the mean and standard deviation. Chi-square test, Fisher exact probability test and one-way analysis of variance were applied to examine the degenerative changes of the sagittal alignment in subjects among different groups. Parameters between subjects with PDS and those without PDS in Group IV were compared using the student t test and chi-square test. The Pearson correlation coefficient was used to analyze the relationships between the variations. The significance threshold was set at 5% (*P* < 0.05).

## Results

### Demographics

A total of 270 subjects (154 females and 116 males), with a mean age of 58.6 years ranging from 20 to 87 years, met the inclusion criteria and were included in the final analysis.

The subjects were distributed into two groups; those with PDS and those without PDS. Those without PDS were in turn distributed into four age groups; Group I (*N* = 35) were young adults (aged 20–35 years), Group II (*N* = 41) were adults (aged 36–50 years), Group III (*N* = 78) were middle-aged (aged 51–65 years) and Group IV (*N* = 71) were elderly patients (aged >65 years). Subjects with PDS (*N* = 45) included 15 males and 30 females with a mean age of 71.9 years (range 66–87 years). Group I included 17 males and 18 females with a mean age of 28.8 years. Group II included 16 males and 25 females with a mean age of 42.8 years. Group III included 35 males and 43 females with a mean age of 60.2 years. Group IV included 33 males and 38 females with a mean age of 73.0 years.

### Change in spinal alignment in subjects without PDS

The demographics and radiological parameters among Groups I, II, III and IV are compared in Table 1. Among the four groups, there was a stepwise increase in the age, TPA, PT, PI-LL, TK and TLK with increasing grade (all *P* < 0.001). There was also a stepwise increase in the LDI among the groups (*P* < 0.05). The PI and sex distribution were identical among the four groups (all *P* > 0.05).

**Table 1:** Sagittal alignment in subjects without PDS.

Parameters	Group I	Group II	Group III	Group IV	*P* value
N	35	41	78	71	
Age (years)	28.8 ± 4.3	42.8 ± 4.7	60.2 ± 3.7	73.0 ± 5.5	0.000**
Sex (M: F)	17:18	16:25	35:43	33:38	0.841
TPA (°)	4.2 ± 4.6	4.4 ± 5.0	10.0 ± 6.1	12.3 ± 7.0	0.000**
PI (°)	40.4 ± 5.1	40.2 ± 6.0	41.2 ± 6.2	40.5 ± 6.4	0.834
PT (°)	7.4 ± 5.4	9.3 ± 5.8	12.9 ± 6.5	15.4 ± 7.7	0.000**
SS (°)	32.7 ± 6.7	30.3 ± 7.3	27.6 ± 8.8	24.5 ± 7.9	0.000**
LL (°)	45.9 ± 9.8	42.1 ± 11.7	36.6 ± 15.9	36.1 ± 11.7	0.000**
PI-LL (°)	−5.5 ± 8.6	−1.9 ± 10.0	4.6 ± 13.3	4.8 ± 13.0	0.000**
L4-S1 (°)	31.7 ± 7.7	30.5 ± 7.3	30.8 ± 11.6	30.4 ± 10.3	0.926
LDI	0.7 ± 0.2	0.7 ± 0.2	1.2 ± 1.7	1.7 ± 4.4	0.037*
TLK (°)	6.3 ± 8.0	8.0 ± 8.4	14.1 ± 10.9	17.8 ± 13.1	0.000**
TK (°)	21.6 ± 12.6	22.1 ± 14.9	26.1 ± 13.3	31.9 ± 15.6	0.000**

PDS, primary degenerative scoliosis; TPA, T1 pelvic angle; PI, pelvic incidence; PT, pelvic tilt; SS, sacral slope; LL, lumbar lordosis; PI-LL, pelvic incidence minus lumbar lordosis; LDI, lordosis distribution index; TLK, thoracolumbar kyphosis; TK, thoracic kyphosis.

** Indicates *P* < 0.01.

* Indicates *P* < 0.05.

**Figure 2:**
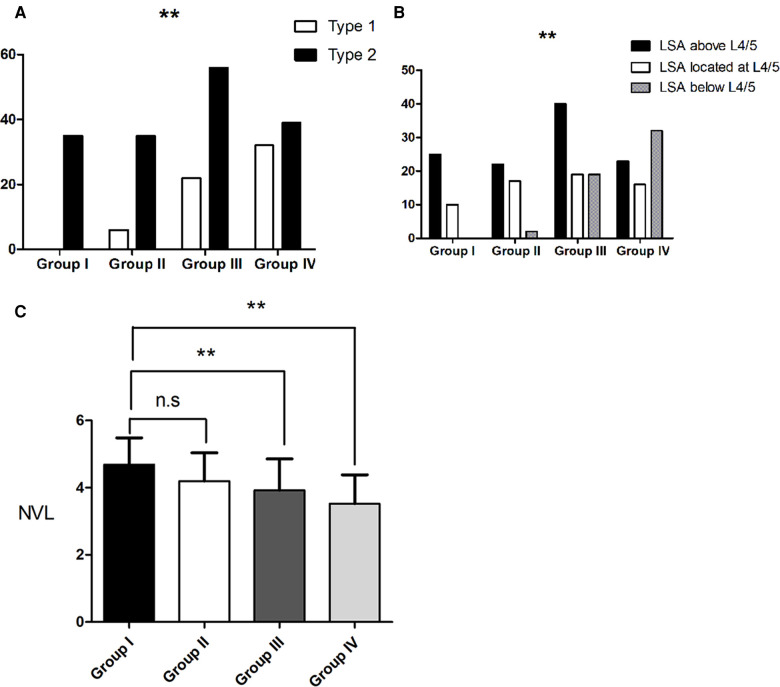
Change in spinal alignment in subjects without primary degenerative scoliosis (**A)**. The proportion of type 1 subjects increased with age among the groups. (**B**). LSA tends to be lower with an increase in age. (**C**). There was also a stepwise decrease in the NVL among the groups. Group I were young adults (aged 20–35 years), Group II were adults (aged 36–50 years), Group III were middle-aged (aged 51–65 years) and Group IV were elderly patients (aged >65 years). LSA, lumbar sagittal apex; NVL, number of vertebrae included in the lordosis; ** indicates *P* < 0.01.

In Group I, all subjects were Roussouly type 2. In Group II, 14.6% of the subjects were type 1, while 85.4% were type 2. In Group III, 28.2% of the subjects were type 1, while 71.8% were type 2. In Group IV, 45.1% and 54.9% of the subjects were type 1 and type 2, respectively. The proportion of type 1 subjects increased with age among the groups (*P* < 0.001) (Figure 2A). In Group I, the number of subjects with the LSA above L4/5 was 25 and for 10 it was located at L4/5, whereas none had the LSA below L4/5. In Group IV, 23 subjects had the LSA above L4/5, for 16 the LSA was located at L4/5 and for 32 the LSA was below L4/5. The LSA tended to be lower in the spine with increasing age (*P* < 0.001) (Figure 2B). There was also a stepwise decrease in the NVL among the groups (*P* < 0.001) (Figure 2C).

### Change in spinal alignment in subjects without PDS who displayed roussouly type 1

**Figure 3:**
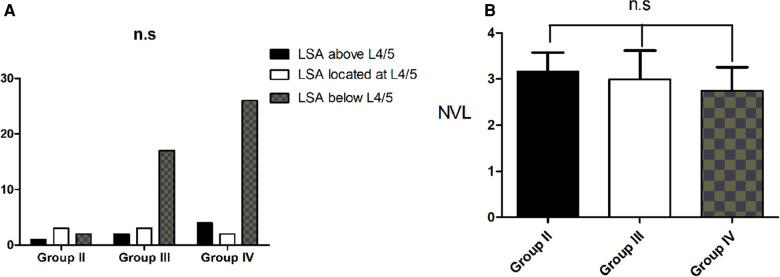
Change in spinal alignment in subjects without primary degenerative scoliosis who displayed roussouly type 1. (**A**). There was no statistical difference among the four groups for the LAS distribution. (**B**). There was no statistical difference among the groups for the NVL distribution.

Two subjects displayed type 1 in Group II, 22 subjects in Group III and 32 subjects in Group IV. There was a stepwise increase in the age, TPA, LDI, TLK and TK from Group I through to Group IV (*P* < 0.05). The sex distribution, PI, PT, SS, LL, L4–S1 and PI-LL were identical among the four groups (all *P* > 0.05) (Table 2). There was also no statistical difference among the four groups for the NVL or LAS distribution (both *P* > 0.05) (Figures 3A,B).

**Table 2:** Change in spinal alignment in subjects without PDS who displayed roussouly type 1.

Parameters	Group II	Group III	Group IV	*P* value
N	6	22	32	
Age (years)	44.0 ± 4.4	61.1 ± 3.4	73.5 ± 5.9	0.000**
Sex (M: F)	3:3	12:10	17:15	1.000
TPA (°)	4.7 ± 2.2	10.6 ± 6.7	12.9 ± 8.4	0.048*
PI (°)	37.3 ± 5.5	37.6 ± 6.1	38.8 ± 7.0	0.763
PT (°)	10.5 ± 3.6	14.5 ± 7.6	16.3 ± 9.2	0.280
SS (°)	25.5 ± 3.4	22.7 ± 7.9	21.8 ± 9.0	0.603
LL (°)	36.7 ± 10.7	29.3 ± 15.1	35.2 ± 16.6	0.336
PI-LL (°)	0.7 ± 9.8	8.3 ± 13.0	3.6 ± 15.9	0.367
L4-S1 (°)	32.3 ± 8.3	36.7 ± 9.9	36.3 ± 9.9	0.617
LDI	0.9 ± 0.1	1.5 ± 0.7	2.8 ± 6.5	0.001**
TLK (°)	14.5 ± 9.0	24.0 ± 13.1	27.7 ± 11.0	0.041*
TK (°)	18.3 ± 13.9	22.1 ± 13.9	37.7 ± 16.1	0.000**

Please refer to Table 1 for definitions of the terms.

** Indicates *P* < 0.01.

* Indicates *P* < 0.05.

### Change in spinal alignment in subjects without PDS who displayed roussouly type 2

**Figure 4:**
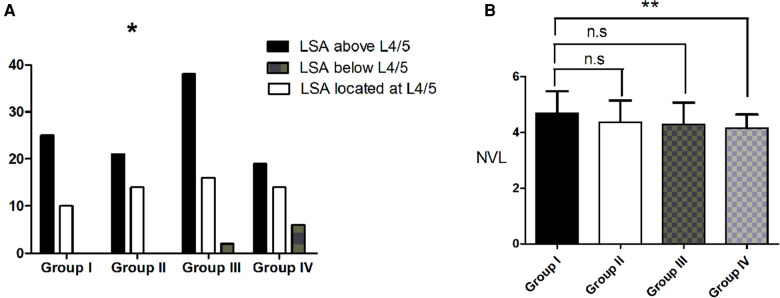
Change in spinal alignment in subjects without primary degenerative scoliosis who displayed roussouly type 2. (**A**). LSA tended to become lower with an increase in age. (**B**). There was also a stepwise decrease in the NVL among the groups. * indicates *P* < 0.05.

There were 35 subjects who displayed type 2 in Group I, 35 subjects in Group II, 56 subjects in Group III and 39 subjects in Group IV. There was a stepwise increase in the age, TPA, PT and PI-LL from Group I through to Group IV (*P* < 0.001). There was also a tendency for an increase in the TLK among the four groups (*P* < 0.05). There was a stepwise decrease in the SS, LL and L4–S1 among the four groups (all *P* < 0.05). The sex distribution, PI, LDI and TK were identical among the four groups (all *P* > 0.05) (Table 3). The LSA tended to be lower in the spine with increasing age (*P* < 0.05) (Figure 4A). There was also a stepwise decrease in the NVL among the groups (*P* < 0.05) (Figure 4B).

**Table 3:** Change in spinal alignment in subjects without PDS who displayed roussouly type 2.

Parameters	Group I	Group II	Group III	Group IV	*P* value
N	35	35	56	39	
Age (years)	28.8 ± 4.3	42.6 ± 4.7	59.9 ± 3.8	72.5 ± 5.3	0.000**
Sex (M: F)	17:18	13:22	23:33	18:21	0.768
TPA (°)	4.2 ± 4.6	4.4 ± 5.4	9.7 ± 5.8	11.9 ± 5.8	0.000**
PI (°)	40.4 ± 5.1	40.7 ± 6.0	42.6 ± 5.8	42.0 ± 5.4	0.225
PT (°)	7.4 ± 5.4	9.1 ± 6.1	12.4 ± 6.0	14.7 ± 6.4	0.000**
SS (°)	32.7 ± 6.7	31.1 ± 7.5	29.6 ± 8.4	26.8 ± 6.0	0.005**
LL (°)	45.9 ± 9.8	43.0 ± 11.7	39.4 ± 15.4	36.1 ± 9.4	0.000**
PI-LL (°)	−5.5 ± 8.6	−2.3 ± 10.2	3.1 ± 13.3	5.9 ± 10.1	0.000**
L4-S1 (°)	31.7 ± 7.7	30.2 ± 7.2	28.5 ± 11.5	25.5 ± 7.7	0.006**
LDI	0.7 ± 0.2	0.7 ± 0.1	1.0 ± 2.0	0.7 ± 0.2	0.529
TLK (°)	6.3 ± 8.0	6.9 ± 7.9	10.3 ± 6.8	9.7 ± 8.3	0.042*
TK (°)	21.6 ± 12.6	22.7 ± 15.2	27.6 ± 12.8	27.2 ± 13.6	0.100

Please refer to Table 1 for the definitions of the terms.

** Indicates *P* < 0.01.

* Indicates *P* < 0.05.

### Sagittal alignment comparison between subjects with and without PDS in group iv

**Figure 5:**
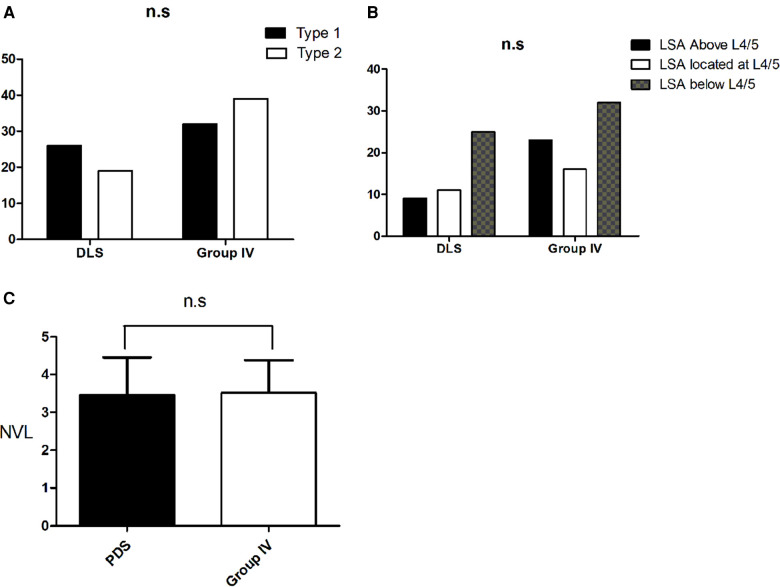
Sagittal alignment comparison between subjects with primary degenerative scoliosis and subjects in Group IV. (**A**). The Roussouly-type distribution was identical between the two groups (**B**). There was no statistical difference between the two groups for the LSA distribution. (**C**). There was also no statistical difference between the two groups for the NVL distribution.

All the subjects with PDS were older than 65 years and had a similar age range to that of Group IV, therefore, we compared the sagittal alignment between these two groups (Table 4). The age, sex distribution, PI, L4-S1, LDI and TLK showed no statistical difference between the two groups (all *P* > 0.05). For subjects with PDS, the TPA, PT and PI-LL were greater than for the subjects without PDS in Group IV (all *P* < 0.05), whereas the SS, LL and TK were less (all *P* < 0.05). The Roussouly-type, NVL and LSA distribution were identical between the two groups (all *P* > 0.05) (Figures 5A–C).

**Table 4:** Sagittal alignment comparison between all subjects with PDS and those subjects without PDS who were in group IV.

Parameters	PDS	Group IV	*P* value
N	45	71	
Age (years)	71.9 ± 5.6	73.0 ± 5.5	0.335
Sex (M: F)	15:30	33:38	0.161
TPA (°)	17.5 ± 10.0	12.3 ± 7.0	0.003**
PI (°)	41.7 ± 7.4	40.5 ± 6.4	0.373
PT (°)	20.3 ± 8.2	15.4 ± 7.7	0.002**
SS (°)	20.8 ± 9.5	24.5 ± 7.9	0.025*
LL (°)	27.7 ± 15.8	35.7 ± 13.1	0.006**
PI-LL (°)	14.0 ± 15.5	4.8 ± 13.0	0.001**
L4-S1 (°)	30.9 ± 10.8	30.4 ± 10.3	0.798
LDI	2.2 ± 4.6	1.7 ± 4.4	0.553
TLK (°)	18.3 ± 13.3	17.8 ± 13.1	0.843
TK (°)	23.8 ± 12.5	31.9 ± 15.6	0.004**

Please refer to Table 1 for the definitions of the terms.

** Indicates *P* < 0.01.

* Indicates *P* < 0.05.

### Impact of PDS on Roussouly's sagittal shape classification

In 17 subjects, the AVR of the thoracolumbar curve displayed Nash-Moe degree I, 22 subjects showed degree II and six subjects had degree III. None of the subjects showed degree 0 or degree IV. There was no difference in the LL, L4-S1, LDI, TLK, TK or NVL among these three degree groups (all *P* > 0.05) (Table 5).

**Table 5:** Impact of AVR on sagittal alignment.

AVR	LL	L4-S1	LDI	TLK	TK	NVL
Degree I	33.7 ± 16.5	32.1 ± 10.9	2.9 ± 7.3	16.1 ± 17.8	27.6 ± 13.5	3.8 ± 1.0
Degree II	22.6 ± 14.6	29.3 ± 10.0	1.6 ± 1.2	17.9 ± 12.0	20.8 ± 12.0	3.3 ± 1.0
Degree III	21.7 ± 14.8	33.3 ± 13.8	2.4 ± 2.0	26.3 ± 15.5	24.0 ± 10.3	3.2 ± 0.8
*P* value	0.124	0.615	0.706	0.264	0.245	0.281

AVR, apical vertebra rotation; NVL, number of vertebrae included in the lordosis; for definitions of other terms please refer to Table 1.

When exploring the change in parameters using the coronal Cobb angle, we found that the PT and TPA increased with an increasing TLC Cobb angle, whereas the LL, SS and NVL decreased. Moreover, as shown in Table 6, the LSC Cobb angle positively correlated with the TK, PI-LL and TPA and negatively correlated with LL. The other parameters did not correlate with the TLC Cobb angle or the LSC Cobb angle (*P* > 0.05).

**Table 6:** Correlation between the TLC Cobb angle, the LSC Cobb angle and other parameters.

Cobb angle	LL	L4-S1	LDI	TLK	TK	PI-LL	PT	SS	TPA	NVL
TLC	−0.297*	0.028	0.116	−0.224	0.169	0.250	0.387**	−0.432**	0.299*	−0.318*
LSC	−0.340*	−0.053	0.002	−0.125	0.342*	0.322*	0.274	−0.280	0.310*	−0.099

TLC, thoracolumbar coronal; LSC, lumbar-sacrum coronal; NVL, number of vertebrae included in the lordosis; for definitions of other terms please refer to Table 1.

** Indicates *P* < 0.01.

* Indicates *P* < 0.05.

## Discussion

Restoring the sagittal spinal contour to the normal and original Roussouly shape according to the PI could reduce specific degeneration changes in the spine (5). Knowing the physiological shape of a patient can also help to plan the surgical restoration of a proper sagittal profile, in the belief that such restoration can lead to better functional outcomes and fewer mechanical complications (5, 8, 13, 14). With each Roussouly-type having a specific LSA, IP and NVL (1, 2, 6), this should be taken into consideration when restoring the ideal sagittal profile. Not considering this algorithm has a threefold risk for increased mechanical complications (12, 15, 16). The level of the LSA was also found to be a significant risk factor for proximal junctional kyphosis after adult spinal deformity surgery (17).

Due to our specific selection criteria, the entire analyzed population had a low PI (≤50°). These subjects with a low PI have two possible types: 1 and 2. What would be the original shape of a degenerated type 1? The present study attempted to examine the radiological characteristics of Roussouly types with a PI ≤ 50° to identify whether type 2 could evolve into type 1 with an increase in age.

It was reported that type 2 was the least common category, which accounted for approximately 11% of the normal population in the original study, whereas type 1 accounted for approximately 21%, type 3 accounted for approximately 38% and type 4 accounted for approximately 30% (2). By contrast, type 2 accounted for 13.9% and type 1 for 15.4% of the degenerative population (8). However, neither study showed how the proportions of the different types changed with age. In our study, all subjects were type 2 in Group I, whereas subjects with type 1 represented 14.6% and type 2 accounted for 85.4% in Group II. In Group III, subjects with type 1 represented 28.2%, while type 2 accounted for 71.8%. In Group IV, subjects with type 1 and type 2 represented 45.1% and 54.9%, respectively. There was a stepwise increase in the proportion of type 1 with age, whereas type 2 decreased (*P* < 0.05). This finding suggested that part of type 1 at least may be a regression of type 2.

Life is a kyphosing event. For all the subjects without PDS in this study, there was a stepwise increase in the TPA, PI-LL, PT, TLK and TK with age, whereas LL and the SS decreased. For the type 1 subjects, there was a stepwise increase in the TPA, TLK and TK with age, whereas the LL, PT, SS and PI-LL remained constant among the different age groups. These results were slightly different from a previous study, which reported that there was a stepwise increase in the PT and TLK with age, whereas LL and the SS decreased and TK were identical among the groups for subjects with type 1 (18). A similar phenomenon was found in the subjects with type 2 in our study. This may be due to type 1 being dependent on the shape of LL: short LL with the apex at L5 in a previous study (18), whereas we defined type 1 as a long TLK and a short lumbar lordotic curve. Another reason is the different age groups in the two studies.

There is a consensus that the LSA and IP of type 2 are higher than those of type 1 and there is a greater NVL of type 2 than that of type 1, whereas the LDI of type 2 is lower than that of type 1 (2). In our study, the LSA and IP of subjects without PDS became lower and the NVL decreased with age, with a similar phenomenon being found in the subjects with type 2. There was no statistical difference among the groups for the LAS or IP distribution of subjects with type 1. At the same time, the TLK of subjects with type 2 increased with age. This provides further evidence that some type 1 evolved from type 2.

What could be the original shape of a degenerated type 1? A prior study reported that the answer is probably different in the case of pure TLK without scoliosis compared to lumbar or thoraco-lumbar scoliosis (13). In the case of scoliosis, the increasing apical rotation may induce a thoracolumbar torsion, flexing a previously flat lordosis in TLK (13). In the case without scoliosis, the original shape was probably a type 1 with a respective increasing TLK and distal hyperlordosis (13). However, some authors described in the literature that even in type 2 subjects without scoliosis, as degeneration progresses, the kyphosing event affects the thoracolumbar area or lumbar area, the L4–S1 angle may increase on a small arch, which can generate a type 1 spine (8), thus this opinion is a little different from the former. That is, the reason why type 2 subjects without scoliosis can become type 1 was increased kyphosis in the thoracolumbar region and increased lordosis of the lower lumbar spine, while type 2 subjects with scoliosis can become type 1 because of the increasing apical rotation. In the present study, there was a tendency for an increase in TLK with age for subjects displaying type 2. However, LL and L4–S1 decreased in this group, and the main reason may be that some type 2 subjects become type 1, the kyphosing event continuing to affect the lumbar area in the rest of type 2 subjects as degeneration progresses. Of course, part of the degenerated type 1 was an original type 1. We found that there was a stepwise increase in TLK and TK with an increase in age for the type 1 subjects. There was no statistical difference in sagittal alignment besides the TPA, PT and PI-LL between the PDS and Group IV, who had the same age in this study. This suggests that PDS only exacerbates the sagittal imbalance. Furthermore, the AVR made no difference to the sagittal alignment in PDS. We believe that the reason was that there was very little rotation of the apical vertebra in PDS and only six subjects displayed Grade III in the present study. Moreover, we found that neither the TLC Cobb angle nor the LSC angle correlated with TLK. Therefore, we believe that PDS had little effect on whether type 2 becomes type 1. This is very similar to what was found in adolescent idiopathic scoliosis (19) and adult scoliosis (20), where the curve type was not associated with a specific pattern of sagittal morphology.

On the basis of the specific geometry of the type 1 back, degenerative patterns associated with the worsening of TLK were hypothetically proposed: degenerative discopathy in the thoracolumbar kyphosis area, retrolisthesis in the junctional area and joint facet arthritis in the hyperlordosis area (5). There is a strong belief that the correct sagittal shape must be restored with surgery to match the physiological or theoretical one. Surgical treatment of TLK remains unclear for patients with a low PI. On the basis of this new sagittal evaluation, the strategy of balance restoration in type 1 (TLK combined with a low PI) points to two treatment options: maintain a type 1 or transform into type 2. Distinguishing between a false and an original type 1 is of great importance for the surgeons. We propose that the following points may help to distinguish the two. For the original type 1, severity of degeneration, including degenerative discopathy, retrolisthesis, joint facet arthritis and degenerative paravertebral muscles, are less than for the false one. Additionally, no obvious tenderness is present in the thoracolumbar region in the original type 1 subjects.

There were some limitations in our study. First, this is a cross-sectional study which cannot precisely ascertain the evolution of the degenerations over time because the evaluated spinal and pelvic parameters were fixed in time. Longitudinal cohort studies are thus warranted to confirm the actual degenerative changes. Second, determination of Roussouly types 1 and 2 using a cutoff value of PI of ≤50° is also arbitrary. There were some studies that set a cutoff value of PI of <45° to determine the Roussouly types 1 and 2 (6). Finally, the retrospective design and the small sample size likely affected the strength of the statistical analysis of the study. More investigations are needed to prove our hypothesis.

## Conclusion

Subjects who display Roussouly type 2 could evolve into the type 1 shape as the deterioration of the sagittal spinal alignment progresses with age. PDS had little effect on whether type 2 becomes type 1. Sagittal shape recognition will help restore the appropriate theoretical shape through surgery, which can eventually lead to better surgical outcomes.

## Data Availability

The original contributions presented in the study are included in the article/Supplementary Material, further inquiries can be directed to the corresponding author/s.
